# The burden and control of congenital syphilis: a need for a comprehensive strategy

**DOI:** 10.2478/abm-2023-0037

**Published:** 2023-08-07

**Authors:** Editorial Office of Asian Biomedicine

**Affiliations:** Faculty of Medicine, Chulalongkorn University, Bangkok 10330, Thailand

Syphilis is caused by the spirochete bacterium *Treponema pallidum*. If inadequately treated during pregnancy, the spirochete can lead to congenital syphilis (CS), which can give rise to subsequent severe sequelae or fetal, neonatal, or infant death [1].

The burden of CS varies. Recent estimates suggest that CS has decreased worldwide between 2012 and 2016 [2]. However, in some countries such as the United States, reported cases of CS have increased since 2012 [2, 3].

CS is acquired through transplacental transmission of spirochetes. Pregnant mothers with untreated primary or secondary syphilis are at the highest risk of transmitting syphilis to the fetus during pregnancy. Vertical transmission of spirochetes to offspring commonly occurs from mothers who are inadequately treated or not treated at all [4]. Since 2007, the World Health Organization has advocated the global elimination of mother-to-child transmission of *T. pallidum* aiming at controlling CS, which can cause significant severe sequelae and burden to individuals and the society [5].

Manifestations of early CS are varied, and diagnosis in newborn infants is difficult [1]. It is therefore important to recognize CS in infants and young children with unexplained growth problems and biochemical and hematological abnormalities [6]. All infants born to mothers who have reactive nontreponemal and treponemal tests for syphilis during pregnancy, as well as infants and children with clinical findings compatible with CS, should be initially evaluated. These can be done using existing screening tests such as Venereal Disease Research Laboratory (VDRL) test and rapid plasma reagin titer [6]. Direct fluorescent antibody staining, darkfield microscopy, and/or polymerase chain reaction (PCR) of nasal discharge and/or swabs of skin lesions should be performed if available. A diagnosis of CS may be established by the demonstration of serologic reactions typical of syphilis in relation to mother's serology, the detection of *T. pallidum* by darkfield microscopy, or PCR of infected body fluids or lesions; the demonstration of *T. pallidum* is achieved by special stains or histopathologic examination [6, 7, 8].

Kulsirichawaroj and Lumbiganon [9] report, in a large study in this volume, that the incidence of CS in a university hospital is high. Factors associated with CS include inadequate screening and treatment of maternal syphilis and preterm birth. They advocate a systematic approach to improvement of antenatal care. This would require efforts toward evidence-based, interprofessional strategies. A collaborative perinatal/neonatal preventive approach to the care of pregnant women may reduce the current trends of CS in countries worldwide. Strategies prioritizing early identification and treatment of at-risk neonates are necessary to reduce– and eliminate – the devastating long-term consequences of the conditions in vulnerable populations.
